# Comparative efficacy of two primary care interventions to assist withdrawal from long term benzodiazepine use: A protocol for a clustered, randomized clinical trial

**DOI:** 10.1186/1471-2296-12-23

**Published:** 2011-04-20

**Authors:** Caterina Vicens, Isabel Socias, Catalina Mateu, Alfonso Leiva, Ferran Bejarano, Ermengol Sempere, Josep Basora, Vicente Palop, Marta Mengual, Jose Luis Beltran, Enric Aragonès, Guillem Lera, Silvia Folch, Josep Lluís Piñol, Magdalena Esteva, Miguel Roca, Arturo Arenas, María del Mar Sureda, Francisco Campoamor, Francisca Fiol

**Affiliations:** 1 Son Serra- La Vileta Health Care Centre, Balearic Mental Health Research Group (rediAPP-Primary Care Research Network), Balearic Health service-IbSalut, Mallorca, Spain; 2 Manacor Health Care Centre, Balearic Mental Health Research Group (rediAPP-Primary Care Research Network), Balearic Health service-IbSalut, Mallorca, Spain; 3 Primary Care Research Unit of Mallorca, CAIBER, Balearic Health service-IbSalut, Mallorca, Spain; 4 Camp de Tarragona Primary Care Department, Reus-Tarragona Research Group (rediAPP-Primary Care Research Network), Catalunya Health service-CatSalut, Tarragona, Spain; 5 Paterna Health Care Centre, Balearic Mental Health Clinical Group (rediAPP-Primary Care Research Network), Valencia Health service- Agència Valenciana de Salut, Valencia, Spain; 6 Biomedical Research Institute Pere Virgili, CIBEROBN (Ciber obesity and nutrition), Carlos III Health Institute, Tarragona, Spain; 7 Hospital la Ribera Primary Care Department, Balearic Mental Health Clinical Group (rediAPP-Primary Care Research Network), Valencia Health service- Agència Valenciana de Salut, Valencia, Spain; 8 Altabix Health Care Centre, Valencia Health service- Agència Valenciana de Salut, Valencia, Spain; 9 Constantí Health Care Centre, Reus-Tarragona Research Group (rediAPP-Primary Care Research Network), Catalunya Health service-CatSalut, Tarragona, Spain; 10 Hospital la Ribera, Conselleria de Sanitat Comunidad Valenciana, Valencia, Spain; 11 Tarragona-Reus Health Care Centre, Reus-Tarragona Research Group (rediAPP-Primary Care Research Network), Catalunya Health service-CatSalut, Tarragona, Spain; 12 Reus-Altebrat Health Care Centre, Reus-Tarragona Research Group (rediAPP-Primary Care Research Network), Catalunya Health service-CatSalut, Tarragona, Spain; 13 Primary Care Research Unit of Mallorca, Balearic Health services Research and Cancer Group (rediAPP-Primary Care Research Network), Balearic Health service-IbSalut, Mallorca, Spain; 14 Institut Universitari d'Investigació en Ciències de la Salut (IUNICS), Balearic Mental Health Research Group (rediAPP-Primary Care Research Network), University of Balearic Islands, Mallorca, Spain; 15 Marines Health Care Centre, Balearic Mental Health Clinical Group (rediAPP-Primary Care Research Network), Balearic Health service-IbSalut, Mallorca, Spain; 16 Son Cladera Health Care Centre, Balearic Mental Health Clinical Group (rediAPP-Primary Care Research Network), Balearic Health service-IbSalut, Mallorca, Spain; 17 Son Espases Hospital, Pharmacy Department, Mallorca, Spain

## Abstract

**Background:**

Although benzodiazepines are effective, long-term use is not recommended because of potential adverse effects; the risks of tolerance and dependence; and an increased risk of hip fractures, motor vehicle accidents, and memory impairment. The estimated prevalence of long-term benzodiazepine use in the general population is about 2,2 to 2,6%, is higher in women and increases steadily with age. Interventions performed by General Practitioners may help patients to discontinue long-term benzodiazepine use. We have designed a trial to evaluate the effectiveness and safety of two brief general practitioner-provided interventions, based on gradual dose reduction, and will compare the effectiveness of these interventions with that of routine clinical practice.

**Methods/Design:**

In a three-arm cluster randomized controlled trial, general practitioners will be randomly allocated to: a) a group in which the first patient visit will feature a structured interview, followed by visits every 2-3 weeks to the end of dose reduction; b) a group in which the first patient visit will feature a structured interview plus delivery of written instructions to self-reduce benzodiazepine dose, or c) routine care. Using a computerized pharmaceutical prescription database, 495 patients, aged 18-80 years, taking benzodiazepine for at least 6 months, will be recruited in primary care health districts of three regions of Spain (the Balearic Islands, Catalonia, and Valencia). The primary outcome will be benzodiazepine use at 12 months. The secondary outcomes will include measurements of anxiety and depression symptoms, benzodiazepine dependence, quality of sleep, and alcohol consumption.

**Discussion:**

Although some interventions have been shown to be effective in reducing benzodiazepine consumption by long-term users, the clinical relevance of such interventions is limited by their complexity. This randomized trial will compare the effectiveness and safety of two complex stepped care interventions with that of routine care in a study with sufficient statistical power to detect clinically relevant differences.

**Trial Registration:**

Current Controlled Trials: ISRCTN13024375

## Background

Benzodiazepines (BZDs) are used to treat anxiety disorders and sleep disturbance; as adjuvant therapy in patients with schizophrenia, depression, and alcohol withdrawal problems; and as muscle relaxants. BZDs are widely prescribed throughout Spain and in most Western countries [1-3]. The last Spanish National Health Survey [4] showed that 14.3% of subjects had used BZD in the previous 2 weeks, including 29.9% of women over 65 years of age. In 2006, the consumption of defined daily doses (DDDs) per 1,000 inhabitants per day (DHD) in Spain was 69.9 [5], and significant variability among regions of the country was evident [5,6].

Although BZDs are effective in the short term, long-term use is usually not recommended because of potential adverse effects and the risks of development of tolerance and dependence. Prolonged use of BZDs may produce somnolence, memory impairment, and daytime drowsiness; may cause falls resulting in hip fractures; and may result in motor vehicle accidents [7-9]. A meta-analysis evaluating the risks and benefits of long-term BZD use to treat insomnia in adults aged > 60 years concluded that, although sleep quality improved, the magnitude of the effect was small and drug use exposed patients to an increased risk of adverse effects [10]. The number needed to treat (NNT) to achieve an improvement in sleep quality was 13 but the NNT for production of an adverse effect was only 6. In addition, several recent studies have associated the regular use of sedative drugs with increased mortality [11,12]. Thus, Spanish guidelines for the management of patients with anxiety disorders and insomnia in primary care recommend restricting BZD use to no more than 2-4 weeks and that long-term users be regularly followed-up [13-17].

Despite the drawbacks of long-term use of BZDs, such use remains widespread, with many patients being treated for several months, years, or even decades. In some instances, the only reason for continued BZD use is avoidance of withdrawal symptoms. Although long-term use is difficult to define, such use is higher in women than in men and increases with age [2]. For example, a study in Tarragona (Spain) found that 6.9% of primary care attendees had taken BZDs for at least 3 months, including 29% of women older than 65 years [18]. A French study showed that the prevalence of BZD use in the general population was 7.5%, including 14.3% of subjects older than 60 years [19]. Other studies have estimated the prevalence of long-term BZD use in the general population at about 2-2.5%[2,20].

Most BZD prescriptions are written by general practitioners (GPs) [21], who often encounter difficulties in managing withdrawal in patients who become dependent on such agents. The usual clinical protocol for BZD withdrawal is gradual tapering. Several studies have tested the effectiveness of different treatment strategies in decreasing long-term BZD use [22,23]. Gradual tapering approaches may range from minimal interventions to more complex strategies. In a typical minimal intervention [24-26], a GP may advise patients to discontinue long-term BZD use without medical assistance by sending a letter that includes information on the negative consequences of continued use and guidelines on withdrawal. In contrast, systematic discontinuation programs are more intensive in nature; patients gradually discontinue BZD dose under the guidance and follow-up care of a GP [21,27,28]. Addition of either psychological interventions [27-29] or substitutive pharmacotherapy [30] to gradual BZD dose reduction has also been evaluated. All these interventions have been found to be more effective than routine care, but GP involvement and cost vary considerably [23].

We have designed a study protocol to evaluate the effectiveness of two interventions, both implemented in primary care, to withdraw chronic BZD users from such drugs. The first is an educational intervention reinforced by a systematic discontinuation program with follow-up visits, and the second is an educational intervention reinforced by written support; both will be compared with routine care. The study is also designed to evaluate the safety of such interventions, as shown by their effects on symptoms of anxiety and depression and on sleep quality and alcohol consumption.

## Methods/Design

### Design and Settings

**Figure 1:**
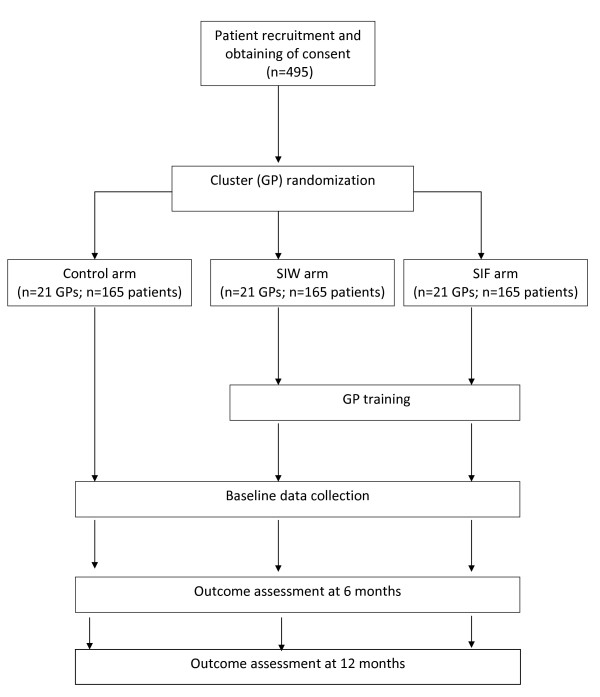
**Flow-Chart of participants**.

The study is designed as a multicenter three-armed clustered randomized clinical trial in primary care settings in three Spanish Primary Care Health Regions, with evaluation at 6 months and blind evaluation at 12 months (Figure 1). Long-term BZD users will be assigned to one of three parallel groups:

a) The first intervention group will receive a structured interview and follow-up visits (SIF).

b) The second intervention group will receive a structured interview and written instructions (SIW).

c) A control group (RC) will receive routine care.

#### Study population

Patients aged 18 to 80 years, who have been taking BZD or related drugs (zopiclone, zolpidem, or zaleplon) for at least 6 months and who do not meet any exclusion criterion will be identified from GP clinical records and invited to participate. Exclusion criteria will include severe depression or anxiety disorder, a psychotic disorder, or a severe personality disorder; current treatment by a psychiatrist; cognitive impairment or advanced neurological disease; consumption of illegal drugs or alcohol abuse; institutionalization; and terminal illness. Other exclusion criteria will be presentation with symptoms of anxiety/depression; a GP view that a subject is likely to be adversely affected by BZD withdrawal; participation in any clinical trial within the previous 3 months; and incapacity or unwillingness to provide written informed consent.

#### Recruitment of GPs and patients

Seventy GPs will be contacted through the research units of each of the three Primary Care Health Districts and invited to participate. Before random allocation to one of the three study arms, each GP must recruit eight patients over a period of 1 month. These patients will be identified from computerized prescription claims databases. Pharmaceutical specialties will include the N05B and N05C groups, and tetrazepam (M03BX07), of the Anatomical, Therapeutic, and Chemical classification system of the World Health Organization (WHO). Each GP will supply a random list of 30 patients; from this list, 8 eligible patients will be selected after eliminating patients who meet exclusion criteria and those who refuse to participate.

#### Random allocation to study arms

After patients give written informed consent, they will be included and GPs will be randomized to one of the three study arms (SIF, SIW or RC group) using a computer-generated block randomization. The randomization code will not be revealed until patient recruitment is complete.

#### Intervention

All subjects in the SIF and SIW groups will undergo an educational individualized interview with their GPs. This standardized interview will cover the following topics:

1) What are BZDs?

2) What happens if an individual takes a BZD for a long time?

3) Why should I stop taking a BZD?

The concepts of dependence, abstinence, and withdrawal syndrome will be discussed, and information on the importance of gradual dose reduction will be given. Patients prescribed BZDs for insomnia will be told how to improve sleep quality. Dose reduction will be discussed and a tailored dose reduction scheme established. In general, the scheme will consist of a 10-25% reduction in daily dose every 2-3 weeks. Patients will be told about withdrawal symptoms, how to recognize them, and what to do if they appear.

After the first interview, patients in the SIF group will be followed up every 2-3 weeks until dose reduction is complete. During each visit, information gathered on previous visits will be reviewed, both to reinforce any positive achievements and to evaluate adverse effects and/or withdrawal symptoms (e.g., tremor, anxiety, insomnia, convulsions, irritability, and/or dizziness). Following the first interview, patients in the SIW group will be given written individualized gradual dose reduction schedules and will be advised to contact the GP if any problems related to the discontinuation process emerge; the study will not schedule any subsequent visit.

Patients in both the SIF and SIW groups who continue to experience withdrawal symptoms will be prescribed a BZD with a longer half-life (diazepam) in place of any current short half-life BZD that is presently prescribed.

Patients allocated in control group will receive routine care for long term users of BZDs.

### Training

GPs assigned to the SIF and SIW groups will attend a workshop 4 hours in duration, to learn how to conduct the structured interview. The workshop will include training in methods of managing BZD withdrawal (an educational standardized interview, provision of individualized patient information, and optimal gradual dose reduction). In addition, GPs assigned to the SIF group will attend a supplementary 30-minute workshop to standardize dose reduction follow-up visits. Training will be provided by collaborative researchers with extensive experience in educating healthcare professionals to manage cessation of BZD use [13].

#### Outcome Assessment

Measures and variables are summarized, with a timeline, in Table 1. Self-declared BZD cessation, anxiety, depression, sleep quality, and alcohol consumption at 6 months will be assessed by the GP. Outcomes at 12 months will be assessed by an external evaluator blinded to the group to which each patient was allocated.

**Table 1:** Measures, variables, and timeline

Instrument	Assessment area	Time(s) of assessments
Sampling form	Inclusion/exclusion criteria	Before randomization

Sociodemographic data form	Sociodemographic data: age, gender, educational level, labor status, marital status, number of persons living in the home, disabled persons under his/her care.	At baseline

Baseline clinical data form	Identification of consumed benzodiazepines	At baseline
	Dosage and duration of benzodiazepines consumed	
	Original reason for taking benzodiazepines	
	Comorbid chronic physical or psychological diseases	

Benzodiazepine Severity of Dependence Scale (SDS)	Severity of benzodiazepine dependence	At baseline

Hospital Anxiety and Depression Scale (HADS)	Symptoms of anxiety and depression	At baseline, 6 and 12 months

Oviedo Sleep Quality Scale (COS)	Sleep quality and features	At baseline, 6 and 12 months

Alcohol consumption form	Alcohol consumption	At baseline, 6 and 12 months

Antidepressant consumption form	Current consumption of antidepressants	At baseline, 6 and 12 months

Current use of benzodiazepine form	Current consumption of benzodiazepines	At baseline, 6 and 12 months

Adverse effects form	Adverse effects related to benzodiazepine withdrawal	At 6 and 12 months

Use of health resources questionnaire	Number of primary care visits related to tapering of benzodiazepines	At 12 months

All outcome assessors and data analysts will be blinded to patient group allocation. To evaluate the effectiveness of blinding, such individuals will be asked to choose the arm to which they believed each patient was assigned (possible answers: intervention, usual care, or unknown). Individuals who respond "intervention" or "usual care" will be asked to indicate what led to that belief.

Outcome assessments will include the following:

##### Primary outcome measure

The primary outcome measure will be BZD cessation at 12 months, defined as self-reported consumption of fewer than 4 doses per month, and confirmed using computerized prescription data over that time period (prescription claims).

##### Secondary outcome measures

The occurrence of symptoms of anxiety and depression will be assessed at 0, 6, and 12 months using the Hospital Anxiety and Depression (HAD) scale [31,32]. The severity of BZD dependence will be assessed at 0, 6, and 12 months using the BZD Severity of Dependence (SDS) Scale [33]. Sleep features will be assessed at 0, 6, and 12 months using the Oviedo Sleep Quality Scale [34]. Alcohol consumption at 0, 6, and 12 months will be self-reported and quantified by standard drink units.

#### Adverse Effects

An adverse event will be defined as any unfavorable or unintended sign, symptom, or disease that could reasonably be associated with discontinuation of BZD. These include tremor, anxiety, insomnia, convulsions, irritability, and dizziness. Physicians will report any withdrawal symptom related to BZD discontinuation to the trial coordinating center, and the data will analyzed by a safety committee. Any serious adverse event (e.g. death, a life-threatening event, inpatient hospitalization or prolongation of existing hospitalization, persistent or significant disability/incapacity) [35] in any patient in either intervention group during the course of the study will be reported to the ethics committee.

#### Statistical Analysis

##### Sample size

We wish to ensure that the clinical trial has adequate statistical power to detect a clinically significant two-tailed difference of at least 20% and 15% BZD cessation rates at 1 year in the SIF and SIW groups, respectively, compared with the usual care group. Assuming 25% loss to follow-up, we estimate that 129 subjects are needed in each arm.

We expect a 0.04 intra-class correlation coefficient [36], yielding a 1.28 cluster design effect. Thus, the target sample size for each group is 165 patients, or a total of 495 patients.

##### Analysis strategy

We will test for significant differences among the baseline characteristics of the control group and the two intervention groups. We will perform descriptive analysis, with continuous variables summarized using means and standard deviations for normal distributions, and by medians and the 25^th ^and 75^th ^percentiles for non-normal distributions.

All data analyses will involve intention-to-treat populations (i.e., all randomized patients, regardless of participation in any treatment session). This approach reduces the bias that may occur when participants not receiving assigned treatments are excluded from analysis. All tests will be two-sided, and α-values of 0.05 will be considered statistically significant.

We will compare the proportions of patients in each group discontinuing BZDs at 12 months against the usual null hypothesis of no difference between proportions. We will use the Chi-squared test, taking into account the "variance inflation factor" of the physician cluster and intraclass correlation coefficient. We will also calculate 95% confidence intervals to assess the clinical significance of interventions.

In multivariate analysis, we will adjust for potential confounders, if any, using a logistic regression model.

We will estimate relative and absolute risk reduction and the number needed to treat, defined as the estimated number of patients who need to be treated with the intervention (rather than routine care) for one additional patient to be controlled.

We will determine the safety of interventions by comparing levels of anxiety, depression, and sleep quality at 12 months among the three groups, using the Chi- squared test and Student's t-test, corrected by VIF and intraclass correlation coefficient. The proportion of patients with serious adverse events related to BZD discontinuation will also be compared.

All estimates will include 95% confidence intervals. The number needed to treat will be calculated as the reciprocal of the difference between the proportion of patients controlled in each intervention group and the control group.

#### Ethical approval

Our study protocol has been approved by the Primary Care Research Committee and the Mallorca Ethical Committee of Clinical Research (IB 1146/09 PI).

### Limitations

Physicians assigned to the control group may be aware that some interventions can be effective in terms of BZD withdrawal. Thus, the decision by a GP to participate in this study may be associated with a greater motivation to facilitate BZD withdrawal. GPs allocated to usual care may therefore be more liable to discontinue patients from long-term BZD use than will be other GPs. Thus, even patients receiving usual care may be influenced by participation in a clinical trial (the Hawthorne effect).

Clinical trials, in which patients are randomized to an interventional or control group, with the same GP assisting both groups, usually suffer from contamination bias. We have therefore designed a clustered randomized trial, to avoid contamination at the GP level. However, if GPs allocated to different study arms are working in the same Healthcare Centre, a contamination bias may arise if control GPs learn about and deliver the intervention. Were this to occur, the expected differences between groups would decrease.

In clustered clinical trials, in which patient inclusion occurs after randomization, the refusal of a significant number of interventional patients to participate may introduce a selection bias. All patients in our study will be included before randomization to avoid such bias, and baseline characteristics will be compared to guarantee that important factors are balanced across treatment groups.

## Discussion

Patients, especially the elderly, would clearly benefit from BZD withdrawal, in that the risks of falls and cognitive impairment would drop, as would the excess mortality rates observed in patients taking anxiolytic drugs [11,12].

Several studies have assessed the effectiveness of strategies used to discontinue patients from long-term BZD use. However, the various interventions differed in methodology, overall effectiveness, and cost-effectiveness. For example, interventions involving the sending of letters to long-term BZD users had adequate sample sizes and were cost-effective, but only one in five patients ceased BZD use. Gradual discontinuation interventions are much more effective, with variable cessation rates, but such trials have usually had small sample sizes. Provision of psychological support was somewhat more effective than was gradual tapering alone, but professional time requirements and costs were much higher [22,23].

A maximally effective withdrawal strategy, at minimal cost and with a low need for professional time, is especially important in primary care settings because of budgetary limitations and the small amount of GP time available per consultation. We expect that the combination of an educational intervention (reinforced by written information) and an individually tailored tapering program will achieve a discontinuation rate not much lower than that of a systematic discontinuation program with follow-up visits, and will consume less professional time.

Despite the increase in research on interventions effective to cause BZD use cessation, it may be difficult to achieve a high rate of cessation, reflecting the challenge that our objective poses to GPs. This study has therefore been designed to compare whether such interventions are effective and safe compared with routine care.

## Competing interests

The authors declare that they have no competing interests.

## Authors' contributions

CV, CM, IS, FF, AL, FB, ES and ME collectively drafted the study protocol and sought funding and ethical approving. GL, VP, JB, JB, MM, SF and JP are responsible of the management of the trial. All authors have read the draft critically, to make contributions, and have approved the final manuscript. CV is its guarantor.

## Pre-publication history

The pre-publication history for this paper can be accessed here:

http://www.biomedcentral.com/1471-2296/12/23/prepub

## References

[B1] Rayon P Montero D Santamaría B Benzodiazepine consumption in Spain Eur J Clin Pharmacol 1997 52 321 3 10.1007/s00228-997-4015-9 9248774

[B2] Zandstra SM Furer JW Van de Lisdonk EH Van't M Hof Bor JHJ Van Weel C Zitman FG Different study criteria affect the prevalence of benzodiazepine use Soc Psychiatr Epidemiol 2002 37 139 44 10.1007/s001270200006 11990011

[B3] García del Pozo J Abajo F Iglesias Carvajal A García-Pando Montero D Corominas Madurga M Sanz García del Pozo V The Use of Ansiolytic and Hypnotic Drugs in Spain (1995-2002) Rev Esp Salud Publica 2004 78 379 87 15293958 10.1590/s1135-57272004000300007

[B4] Encuesta Nacional de Salud de España 2006 monografía en Internet 2008 Madrid: Ministerio de Sanidad y Consumo http://www.msc.es/estadEstudios/estadisticas/encuestaNacional/encuestaNac2006/UtilizacionServiciosSanitariosAbsoluto.xls accessed 2nd February 2011

[B5] Observatorio del Uso de Medicamentos de la AEMPSmonografía en Internet Madrid: Agencia Española de Medicamentos y Productos Sanitarios http://www.aemps.es/profHumana/observatorio/docs/ansioliticos-hipnoticos.pdf Accessed 2nd february 2011

[B6] Vicens C Sempere E Palop V Moral A Zaforteza M Prat A Consumption of antidepressants and benzodiazepines in our Communities II Congress of the Catalan, Valencian and Balearic Societies of Family and Community Medicine 2008 12 2 Castellon 3 Revista Valenciana de Medicina Familiar

[B7] Herings RM Stricker BH De Boer A Bakker A Sturmans F Benzodiazepines and the risk of falling leading to femur fractures. Dosage more important than elimination half-life Arch Intern Med 1995 155 1801 7 10.1001/archinte.155.16.1801 7654115

[B8] Wangs PS Bohn RL Glynn RJ Mogun H Avorn J Hazardous benzodiazepine regimens in the elderly: effects of a half-life, dosage, and duration on risk of hip fracture Am J Psychiatry 2001 158 892 8 11384896 10.1176/appi.ajp.158.6.892

[B9] Neutel CI Perry S Maxwell C Medication use and risk of falls Pharmacoepidemiol Drug Safety 2002 11 97 104 10.1002/pds.686 11998544

[B10] Glass J Lanctot K Herrman N Sproule A Busto U Sedative hypnotics in older people with insomnia: meta-analysis of risks and benefits BMJ 2005 331 1169 10.1136/bmj.38623.768588.47 16284208 PMC1285093

[B11] Mallon L Broman JE Hetta J Is usage of hypnotics associated with mortality? Sleep Medicine 2009 10 279 86 10.1016/j.sleep.2008.12.004 19269892

[B12] Belleville G Mortality hazard associated with anxiolitic and hypnotic drug use in the National Population Health Survey Can J Psychiatry 2010 55 558 67 20840803 10.1177/070674371005500904

[B13] Ministerio de Sanidad y Consumo (MSC) Guía de Prescripción Terapéutica (GPT) Adaptación española de la 51°ed. del British National Formulary (BNF) 1°ed. Española 2006 Barcelona: Pharma Editores S.L

[B14] Grupo de Trabajo de la Guía de Práctica Clínica para el Manejo de Pacientes con Insomnio en Atención Primaria 2009 Guías de Práctica Clínica en el SNS: UETS 5 1 Guía de Práctica Clínica para el Manejo de Pacientes con Insomnio en Atención Primaria. Plan de Calidad para el Sistema Nacional de Salud del Ministerio de Sanidad y Política Social. Unidad de Evaluación de Tecnologías Sanitarias. Agencia Laín Entralgo. Comunidad de Madrid

[B15] Grupo de Trabajo de la Guía de Práctica Clínica para el Manejo de Pacientes con Trastornos de Ansiedad en Atención Primaria 2008 Guías de Práctica Clínica en el SNS: UETS 10 Guía de Práctica Clínica para el Manejo de Pacientes con Trastornos de Ansiedad en Atención Primaria. Plan de Calidad para el Sistema Nacional de Salud del Ministerio de Sanidad Unidad de Evaluación de Tecnologías Sanitarias. Agencia Laín Entralgo. Comunidad de Madrid

[B16] National Collaborating Centre for Mental Health Depression. The treatment and management of depression in adults 2009 London (UK): National Institute for Health and Clinical Excellence (NICE) 64 Clinical guideline; no. 90

[B17] National Collaborating Centre for Mental Health Anxiety: management of anxiety (panic disorder, with or without agoraphobia, and generalised anxiety disorder) in adults in primary, secondary and community care 2004 London (UK): National Institute for Health and Clinical Excellence (NICE) 88 Clinical guideline; no. 22 20945576

[B18] Bejarano F Romero Piñol JL Moreso Mora N Gilabert Claver P Luque Brull N López Basora J Gallisa Elevado consumo de benzodiacepinas en mujeres ancianas asignadas a centros de salud urbanos de atención primaria Aten Primaria 2008 40 617 21 10.1016/S0212-6567(08)75695-6 19100149 PMC7713455

[B19] Lagnaoui R Depont F Fourrier A Abouelfath A Begaud B Verdoux H Moore N Patterns and correlates of benzodiazepine use in the French general population Eur J Clin Pharmacol 2004 60 523 9 10.1007/s00228-004-0808-2 15338086

[B20] Simpson RJ Power KG Wallace LA Butcher MH Swanson V Simpson EC Controlled comparison of the characteristics of long term benzodiazepine users in general practice Br J Gen Pract 1990 40 22 26 1969288 PMC1371210

[B21] Vicens C Fiol F Llobera J Campoamor F Mateu C Alegret S Socías I Withdrawal from long-term benzodiazepine use: randomised trial in family practice Br J Gen Pract 2006 56 958 63 17132385 PMC1934057

[B22] Oude RC Vosahar Couvée JE Van Balkom A Mulder P Zitman FG Strategies for discontinuing long-term benzodiazepine use British J Psychiatry 2006 189 213 20 10.1192/bjp.189.3.213 16946355

[B23] Parr JM Kavanagh DJ Cahill L Mitchell G McD Young R Effectiveness of current treatment approaches for benzodiazepine discontinuation: a meta-analysis Addiction 2008 104 13 24 18983627 10.1111/j.1360-0443.2008.02364.x

[B24] Cormack MA Sweeney KG Hughes-Jones H Foot GA Evaluation of an easy, cost-effective strategy for cutting benzodiazepine use in general practice Br J Gen Pract 1994 44 5 8 8312045 PMC1238754

[B25] Bashir K King M Ashworth M Controlled evaluation of brief intervention by general practitioners to reduce chronic use of benzodiazepines Br J Gen Pract 1994 44 408 12 8790654 PMC1238990

[B26] Gorgels WJ Oude RC Vosahar Mol AJ Van de Lisdonk EH Van Balkom AJ Van den Hoogen HJM Mulder J Breteler MH Zitman FG Discontinuation of long-term benzodiazepine use by sending a letter to users in family practice: a prospective controlled intervention study Drug and Alcohol Depend 2005 78 49 56 10.1016/j.drugalcdep.2004.09.001 15769557

[B27] Baillargeon L Landreville P Verreault R Beauchemin JP Grégoire JP Morin CM Discontinuation of benzodiazepines among older insomniac adults treated with cognitive-behavioural therapy combined with gradual tapering: a randomized trial CMAJ 2003 169 1015 1020 14609970 PMC236226

[B28] Oude RC Voshaar Gorgels W Mol AJ Van Balkom AJ Van de Lisdonk EH Breteler MH van den Hoogen HJ Zitman FG Tapering off long-term benzodiazepine use with or without group cognitive-behavioral therapy: three condition, randomized controlled trial Br J Psychiatry 2003 182 498 504 10.1192/bjp.182.6.498 12777340

[B29] Morin CM Bastien C Guay B Radouco-Thomas M Leblanc J Vallieres A Randomized clinical trial of supervised tapering and cognitive behavior therapy to facilitate benzodiazepine discontinuation on older adults with chronic insomnia Am J Psychiatry 2004 161 332 42 10.1176/appi.ajp.161.2.332 14754783

[B30] Denis C Fatséas M Lavie E Auriacombe M Pharmacological interventions for benzodiazepine mono-dependence management in outpatients settings Cochrane Database Syst Rev 2006 10.1002/14651858.CD005194.pub2 16856084

[B31] Zigmond AS Snaith RP The hospital anxiety and depression scale Acta Psychiatr Scand 1983 67 361 370 10.1111/j.1600-0447.1983.tb09716.x 6880820

[B32] Quintana JM Padierna A Esteban C Arostegui I Bilbao A Ruiz I Evaluation of the psychometric characteristics of the Spanish version of the Hospital Anxiety and Depression Scale Acta Psychiatr Scand 2003 107 216 21 10.1034/j.1600-0447.2003.00062.x 12580829

[B33] De las Cuevas C Sanz E De la Fuente JA Padilla J Berenguer JC The Severity of Dependence Scale (SDS) as screening test for benzodiazepine dependence: SDS validation study Addiction 2000 95 245 50 10.1046/j.1360-0443.2000.95224511.x 10723853

[B34] Bobes J González MP Sáiz PA Bascarán MT Iglesias C Fernández JM Propiedades psicométricas del cuestionario Oviedo de sueño Psicothema 2000 12 107 12

[B35] Meyboom RHB Royer RJ Causality classification at pharmacovigilance centers in the European Community Pharmacoepidemiol Drug Safety 1992 1 87 9 10.1002/pds.2630010207

[B36] Campbell M Grimshaw J Steen N Sample size calculations for cluster randomised trials. Changing Professional Practice in Europe Group (EU BIOMED II Concerted Action) J Health Serv Res Policy 2000 5 12 6 10787581 10.1177/135581960000500105

